# Outcomes following surgery for fractured neck of femur in dialysis patients: a 5-year review from a district general hospital in the United Kingdom

**DOI:** 10.1186/s12882-016-0234-6

**Published:** 2016-03-03

**Authors:** Oscar Swift, Anouska Ayub, Suresh Mathavakkannan, Nick de Roeck

**Affiliations:** Department of Renal Medicine, Lister Hospital, East and North Hertfordshire NHS Trust, Stevenage, UK; Department of Trauma and Orthopaedics, Lister Hospital, East and North Hertfordshire NHS Trust, Stevenage, UK

**Keywords:** Dialysis patients, Mortality, Fractured neck of femur

## Abstract

**Background:**

Neck of femur fractures are associated with high mortality and have increased prevalence in dialysis patients. Delays in operating on dialysis patients can occur as a result of logistical or medical issues; it has previously been shown that delays on operating on neck of femur fractures in the general population results in increased mortality.

**Methods:**

Medical records of 27 dialysis patients admitted to a large district general hospital in the UK with a fractured neck of femur between January 2009 and January 2014 were analysed alongside records of 27 age and sex-matched non-dialysis patients. Fisher’s exact test and the unpaired *t* test were applied to data to explore outcomes. Odds ratio was also used to compare mortality between the dialysis and non-dialysis groups.

**Results:**

Thirty-day mortality amongst dialysis patients was 22 %, compared to 7 % in the non-dialysis cohort. One-year mortality amongst dialysis patients was 70 %, compared to 15 % in the non-dialysis cohort (odds ratio 13.7 (3.56–52.4, 95 % confidence interval; *p* = 0.0001)). Average length of survival in dialysis patients overall was 311 days; average length of survival if the patient was operated on within 48 h of admission was 450 days (192–708 days, 95 % confidence interval) and was 224 days (45–402, 95 % confidence interval) if operated on after more than 48 h of admission (*p* = 0.16).

**Conclusions:**

Dialysis patients had higher post-operative mortality than the non-dialysis cohort. Odds ratio for death was significantly greater at one-year in the cohort of dialysis patients compared to the non-dialysis patients. Delay to operation amongst the dialysis patient cohort did not contribute significantly to mortality in this study. The higher rates of coronary artery disease, diabetes mellitus and malignancy may confound mortality amongst patients on dialysis who sustain a fractured neck of femur. Limitations of this study included small patient numbers, data from only one centre being used, and some missing data for certain patients.

## Background

Neck of femur fractures are associated with high mortality in frail and elderly populations. In the UK, approximately 70,000 to 75,000 hip fractures occur each year [1]. 8.2 % of people with a hip fracture die within 30-days [2] and approximately one-third within one-year [1]. Many patients with a neck of femur fracture have multiple co-morbidities, including end stage renal failure (ESRF). 25 % of patients on renal replacement therapy (RRT) are now aged over 70 years; an increase of over 5 % from 2000. The prevalence rates in the UK of dialysis in patients greater than 85 years of age has continued to increase from 983 per million population to 1020 per million population between 2012 and 2013 [3]. This follows a global trend of increasing numbers of older, frailer patients commencing dialysis [4].

The incidence rate of hip fracture in Canadian women aged greater than 65 years with an estimated glomerular filtration rate of less than 15 ml/min per 1.73 m^2^ was 25.8 per 1000 person-years [5], compared to 9.2 per 1000 person-years [6] in all women aged 75–84 years. ESRF is associated with reduced bone mineral density, a known risk factor for fragility fractures [7]. Haemodynamic instability between dialysis sessions [8], cardiovascular disease and diabetic and non-diabetic peripheral vascular disease further increase the likelihood of falls and fractures.

Mortality following all fractures has been shown to be 2.5 times greater in dialysis patients [9]. One-year mortality following hip fractures in dialysis patients has been previously quoted at 64 % [10].

Delays in definitive management of patients with a neck of femur fracture have been shown to result in increased mortality [11]. Delays may occur in dialysis patients as surgery may need to be postponed for pre-operative dialysis and logistical issues may mean that patients require transfer to a hospital with both inpatient dialysis and orthopaedic facilities.

## Methods

27 subjects on dialysis presenting with hip fracture were identified using trauma admission and operating theatre records at Lister Hospital, Stevenage, United Kingdom for the period January 2009–January 2014. 27 age and sex matched non-dialysis fractured neck of femur patients admitted over the same time period with a mean pre-operative serum creatinine of 83 μmol/L (0.94 mg/dL) were also identified.

In the United Kingdom many renal units have a ‘hub and spoke’ model whereby the main renal centre is supported by satellite dialysis facilities at hospitals closer to patients homes. Lister Hospital is the central ‘hub’ unit for a catchment population of 1.2 million. Inpatient care during periods of acute illness (such as following fractured neck of femur) occurs at the ‘hub’ site and as a result patients may require transfer from a satellite hospital for treatment.

Between January 2009 and October 2011, both dialysis and non-dialysis fractured neck of femur were admitted to orthopaedic wards at Lister Hospital, Stevenage. In October 2011 the specialist fractured neck of femur service at Lister Hospital was transferred to Queen Elizabeth II Hospital, Welwyn Garden City in order to facilitate local service reconfiguration. Between October 2011 and January 2014 dialysis patients with a fractured neck of femur were treated at Lister Hospital and had access to general orthopaedic services, but not specialist fractured neck of femur services.

All patient identifiable data was removed prior to analysis. Formal approval from the National Research Ethics Service was not required under national terms of guidance. However, permission was obtained for the project from East and North Hertfordshire National Health Service Trust Clinical Audit team (CA number 8048).

The outcomes analysed in the study were overall mortality and the six standards of care outlined in the British Orthopaedic Association “Blue Book”: The Care of Patients with Fragility Fracture [12]:

1. All patients presenting with hip fracture should be admitted to an acute orthopaedic ward within 4 h of presentation.
2. All patients with hip fractures who are medically fit should have surgery within 48 h of admission, and during normal working hours.
3. All patients with hip fractures should be assessed and cared for with a view to monitoring their risk of developing a pressure ulcer.
4. All patients presenting with a fragility fracture should be managed on an acute orthopaedic ward with routine access to acute orthogeriatric medical support from the time of admission.
5. All patients presenting with fragility fracture should be assessed to determine their need for antiresorptive therapy to prevent future osteoporotic fracture.
6. All patients presenting with a fragility fracture following a fall should be offered multidisciplinary assessment and intervention to prevent future falls.

Fisher’s exact test and the unpaired *t* test were applied to data to explore outcomes. Odds ratio was also used to compare mortality between the dialysis and non-dialysis groups.

## Results and discussion

27 dialysis patients (all receiving haemodialysis) were admitted with a fractured neck of femur during the period January 2009 to January 2014. 67 % patients were male. The average patient age was 75.2 years (range 39–93). 33 % patients were admitted to an acute orthopaedic ward within 4 hours of presentation, compared to a national average of 50 % in 2013 [2].

**Fig. 1:**
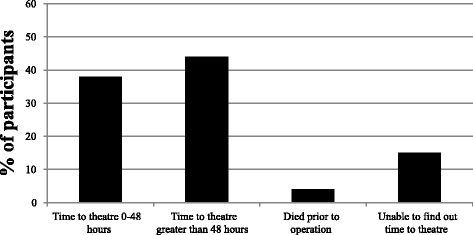
Time from admission to theatre

37 % patients went to theatre within 48 hours of admission, 44 % went to theatre more than 48 h following admission. 4 % died prior to the operation and it was not possible to find out the time from admission to theatre for the other patients (see Fig. 1). Nationally, 86 % of patients underwent surgery within 48 h in 2013 [2].

The average time from admission at the satellite hospital to transfer for this group of patients was 35 hours. The average time from admission at the satellite hospital to operation for this group was 86.3 hours (compared to 71.1 hours for the group overall). All of the patients transferred from a satellite hospital (see Table 1) were operated on more than 48 hours after admission, in contrast to only 31 % of patients admitted directly to Lister Hospital. There were numerous reasons documented in the case notes as to why surgery was delayed (see Table 2).

**Table 1:** Numbers of patients transferred from a satellite dialysis unit

Satellite dialysis unit	Percentage of patients
Luton and Dunstable Hospital	26 %
Bedford Hospital	7 %
Princess Alexandra Hospital, Harlow	4 %
St Albans City Hospital (via Watford General Hospital)	4 %
All satellite dialysis units	41 %

**Table 2:** Documented delays to surgery

Reason for delay	% of patients
Required haemodialysis pre-operatively	22 %
Pre-operative cardiology/ anaesthetic review required	11 %
Initially transferred to QEII Hospital, Welwyn Garden City before transferred to Lister Hospital	7 %
Delay in diagnosis and referral to orthopaedics	7 %
Reason not documented in notes	7 %

56 % of patients underwent fixation with a dynamic hip screw, 11 % underwent fixation with a femoral nail, and 30 % underwent hemiarthroplasty.

**Fig. 2:**
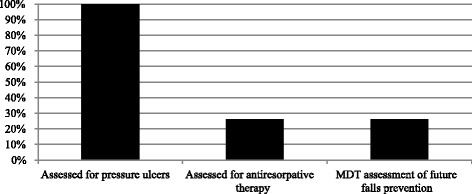
Rates of key assessments for fractured neck of femur patients (as per British Orthopaedic Association guidance)

The renal team reviewed 100 % patients daily during their admission. In addition to this, 19 % patients received additional orthogeriatric input during their inpatient stay. Nationally, 94 % of fractured neck of femur patients received a falls assessment prior to discharge [2], compared to 26 % in this cohort (see Fig. 2).

Outcomes in the dialysis group were also compared with outcomes in 27 age and sex matched non-dialysis group with a mean pre-operative serum creatinine of 83 μmol/L (0.94 mg/dL). Data on pre-operative co-morbidities in both the dialysis group and non-dialysis group is listed in Table 3.

**Table 3:** Co-morbidities of patients with fractured neck of femur in both dialysis and non-dialysis cohorts

	Dialysis patients	Non-dialysis patients	*P* value
Coronary artery disease	26 %	19 %	0.74
Diabetes mellitus	41 %	22 %	0.24
Osteoporosis, osteopaenia or anti-osteoporotic medication	30 %	33 %	0.78
Malignancy	44 %	37 %	0.78
ASA score I (normal healthy patient)	0 %	7 %	
ASA score II (mild systemic disease)	0 %	37 %	
ASA score III (severe systemic disease)	67 %	48 %	
ASA score IV (severe systemic disease that is constant threat to life)	33 %	7 %	

**Fig. 3:**
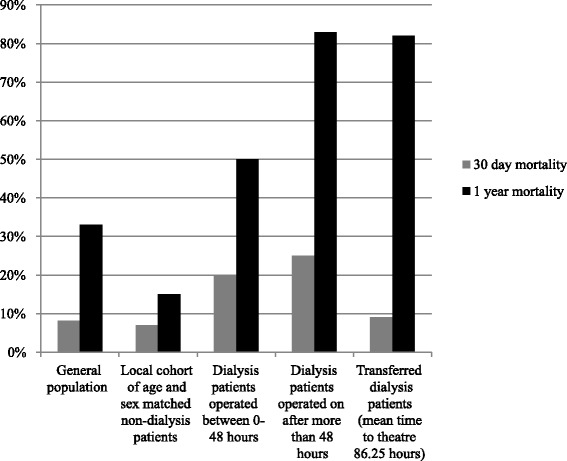
30-day and 1-year mortality rates post fractured neck of femur in dialysis patients

30-day mortality in dialysis patients operated on within 48 hours of admission was 20 % and was 25 % if the patient was operated on after more than 48 hours of admission. Overall 30-day mortality in this cohort was 22 %. The non-dialysis fractured neck of femur patients treated over the same time period had a 30-day mortality of 7 %. 30-day mortality nationally is 8.2 % [2] (see Fig. 3). The odds ratio for death at 30-days in the dialysis group compared to the non-dialysis group was 3.57 (0.65–19.6, 95 % confidence interval; *p* = 0.14).

One-year mortality was 50 % if the patient was operated on within 48 hours of admission and was 83 % if operated on after more than 48 hours of admission. 80 % of patients transferred from a satellite unit died within one year. Overall mortality in this cohort was 70 %. One-year mortality in the local cohort of age and sex matched non-dialysis patients was 15 %. One-year mortality nationally is approximately 33 % [1] (see Fig. 3). The odds ratio for death at one-year in the dialysis group compared to the non-dialysis group was 13.7 (3.56–52.4, 95 % confidence interval; *p* = 0.0001).

When stratified by the American Society of Anaesthesiologists (ASA) score, 30-day mortality was 11 % and 44 % for ASA III and IV patients in the dialysis group, respectively. ASA III patients in the dialysis group had a one-year mortality of 67 %; this value was 78 % in the ASA IV group. The association between patients with an ASA score of III and patients with an ASA score of IV and death within 30-days and one-year was not shown to be statistically significant (*p* = 0.35).

Average length of survival in dialysis patients overall was 311 days; average length of survival if the patient was operated on within 48 h of admission was 450 days (192–708 days, 95 % confidence interval) and was 224 days (45–402, 95 % confidence interval) if operated on after more than 48 h of admission (*p* = 0.16).

Average length of stay in hospital was 33 days, compared to 20 days nationally [2]. 11 % patients were independently mobile at one year.

## Conclusions

One-year mortality in all dialysis patients in the UK in 2013 was 14.5 % [3], which was less than the 70 % described in this cohort. 30-day and one-year mortality rates post fractured neck of femur in our cohort of dialysis patients are greater compared to all fractured neck of femur patients in the UK. Dialysis patients in this cohort had a 13.7 greater chance of death at one-year (3.56–52.4, 95 % confidence interval; *p* = 0.0001) compared to the group of local age and sex matched non-dialysis patients, highlighting the significant burden of dialysis and associated co-morbidities that these patients may have. There was no significant odds ratio reduction in death at 30-days when the dialysis cohort was compared to the non-dialysis cohort. The association between time to operation and length of survival was not statistically significant.

There were numerous postoperative complications in the dialysis cohort that may contribute significantly to mortality (see Table 4). Possible causes of these complications include uraemia, perioperative venous thromboprophylaxis and the physiological stress of surgery (making patients more prone to bleeding), the significant pre-operative co-morbidity of receiving dialysis, higher rates of coronary artery disease, diabetes mellitus and malignancy, and difficulties associated with maintaining a steady euvolaemic state post-surgery.

**Table 4:** Post-operative complications

Post-operative complication	% of patients affected
Upper GI bleed	11
Pneumonia	11
Urosepsis	7
Wound infection	4
Bowel perforation	4
Stroke	4
Pulmonary oedema	4

An improvement in the multidisciplinary team approach of falls assessment is needed for patients receiving haemodialysis with a fractured neck of femur. Dialysis patients may miss out on specialised therapy service input because they are unable to receive standard rehabilitation whilst undergoing dialysis. The involvement of physiotherapists, occupational therapists and orthogeriatricians, working alongside orthopaedic surgeons and renal physicians, may help improve this standard. It is hoped that this will reduce inpatient length of stay, enhance rates of independent mobility at one year and may also result in an improvement in mortality rates.

It must be noted that in this study, there are certain limitations. These include small patient numbers, data from only one centre being used, and some missing data for certain patients. This study applies to a setup where there is a discrepancy in orthopaedic and renal services. However, in the UK, nephrology services are considered a specialised, regional service whereas orthopaedic hip fracture services are available in the majority of district general hospitals.

Whilst the control group were matched for age and sex and had a mean serum creatinine that was within normal limits, risk factors such as history of coronary artery disease, diabetes mellitus and malignancy were higher in the dialysis group, although not significantly so. There were also higher proportions of patients with an ASA score of III or IV in the dialysis group, which reflects higher rates of systemic diseases in this group. Conversely, there were (non-significantly) higher documented rates of osteopaenia, osteoporosis or treatment for osteoporosis in the non-dialysis group, although this may reflect the fact that bisphosphonates are not licensed in advanced renal impairment (see Table 3). Smoking status and body mass index were not available for comparison. The higher rates of coronary artery disease, diabetes mellitus and malignancy may confound mortality amongst patients on dialysis who sustain a fractured neck of femur.

Further work needs to be done using multi-centre data to create a more highly powered study that may help establish a robust protocol for treating dialysis patients with a fractured neck of femur.

## Abbreviations

ASA: American Society of Anaesthesiologists
ESRF: end stage renal failure
RRT: renal replacement therapy
